# Bone turnover in lactating and nonlactating women

**DOI:** 10.1007/s00404-023-07189-0

**Published:** 2023-09-14

**Authors:** Lena Nerius, Mandy Vogel, Uta Ceglarek, Wieland Kiess, Ronald Biemann, Holger Stepan, Jürgen Kratzsch

**Affiliations:** 1 LIFE Leipzig Research Center for Civilization Diseases, University of Leipzig, 04103 Leipzig, Germany; 2 Department of Women and Child Health, Hospital for Children and Adolescents and Center for Pediatric Research, University of Leipzig, 04103 Leipzig, Germany; 3 Institute for Laboratory Medicine, Clinical Chemistry and Molecular Diagnostics (ILM), University of Leipzig, Paul-List-Str. 13-15, 04103 Leipzig, Germany; 4 Department of Obstetrics, University of Leipzig, 04103 Leipzig, Germany

**Keywords:** Lactation, Bone turnover, Breastfeeding, Calciotropic hormones

## Abstract

**Purpose:**

During lactation, bone turnover increases, reflecting the mobilization of Calcium from maternal skeletal stores and resulting in bone loss. However, mechanisms are not yet fully understood, and previous studies have been comparatively small. We aim to assess bone metabolism during lactation by comparing bone-metabolism-related-parameters between large cohorts of lactating and nonlactating women.

**Methods:**

In a retrospective cohort study, we recruited 779 postpartum women and 742 healthy, nonpregnant, nonlactating controls. Postpartum women were examined 3 and 6 months after delivery and retrospectively assigned to either the exclusively breastfeeding (exc-bf) group if they had exclusively breastfed or the nonexclusively breastfeeding (nonexc-bf) group if they had not exclusively breastfed up to the respective visit. Serum levels of PTH, Estradiol, total Calcium, Phosphate, and bone turnover markers (ßCTX, P1NP, Osteocalcin) were compared between the groups.

**Results:**

Bone turnover markers were significantly increased in exc-bf and nonexc-bf women compared with the controls (all *p*s < .001). ßCTX was approximately twice as high in exc-bf women than in the controls. PTH levels were marginally higher in exc-bf (*p* < .001) and nonexc-bf women (*p* = .003) compared with the controls (6 months). Estradiol was suppressed in exc-bf women compared with the controls (*p* < .001, 3 months).

**Conclusion:**

Exc-bf and even nonexc-bf states are characterized by an increase in bone formation and resorption markers. The PTH data distribution of exc-bf, nonexc-bf, and control groups in the underpart of the reference range suggest that lactational bone loss is relatively independent of PTH.

**Supplementary Information:**

The online version contains supplementary material available at 10.1007/s00404-023-07189-0.

## What does this study add to the clinical work

**Table:** 

This retrospective study examines bone turnover during lactation and shows that both bone resorption and bone formation are increased in lactating women compared to healthy, nonlactating, nonpregnant controls. To our knowledge, this is the largest published data that describes bone turnover in the comparison of breastfeeding and nonbreastfeeding women.	

## Introduction

Lactation represents a time of major challenges for the maternal organism. In order to supply the newborn, the lactating mother secretes 300–400 mg of Calcium (Ca) into her breast milk every day [1]. A main source is the maternal bone. Ca is resorbed transiently from the mother’s skeleton, resulting in bone loss that is restored after weaning [2]. Studies found that bone mass decreased by up to 10% in women who breastfed exclusively for 6 months (m) [3, 4]. A main physiological mediator that is attributed to lactational bone loss is Estradiol (E_2_) [5–7]. Lactation causes hypoestrogenemia by suppressing the hypothalamic–pituitary–ovarian axis, thereby inhibiting reproductive function. Different studies have reported significantly lower E_2_ levels in breastfeeding (bf) compared with nonbf postpartum (pp) women and nonlactating, nonpregnant controls [8–14]. In this context, lactation-associated bone loss has been shown to be rather independent of calciotropic hormones, such as Parathyroid Hormone (PTH), which is either normal [15–17] or suppressed [11, 18, 19] in bf women. At the molecular level, bone turnover increases, as reflected by changes in bone turnover markers (BTM). Several studies have consistently found increased levels of bone resorption markers in bf women compared with nonbf women or controls [13, 15, 16, 19–23]. Whereas only a few authors have reported normal levels of bone formation markers [21], the majority of studies have shown significantly increased bone formation in bf women [13, 15, 16, 19, 20, 22, 23]. In a prospective cohort study examining bone turnover during lactation, bone resorption and bone formation, as measured with C-Telopeptide of Type 1 Collagen (ßCTX), Bone-specific Alkaline Phosphatase, Osteocalcin (OC), and Procollagen Type 1 N Terminal Propeptide (P1NP), were significantly increased in women who lactated for 6–8 weeks (wk) compared with bottle-feeding women and controls [13]. However, this study used only a small sample, a trend that applies to most other studies as well, many of which are also outdated. Furthermore, the underlying hormonal mechanisms regulating Ca homeostasis during lactation are not yet fully understood. Findings on concentrations in bf and nonbf women are somewhat contradictory, as reviewed by Kovacs et al. [2]. Therefore, we aim to examine bone metabolism in a large cohort of exclusively bf (exc-bf) and nonexclusively bf (nonexc-bf) pp women as well as healthy, nonpregnant, nonlactating controls using the most current BTM ßCTX, P1NP, and OC, as well as serum PTH, E_2_, total Ca, and Phosphate (P) to provide a representative update on bone metabolism during lactation.

## Methods

### Study design and population

**Fig. 1:**
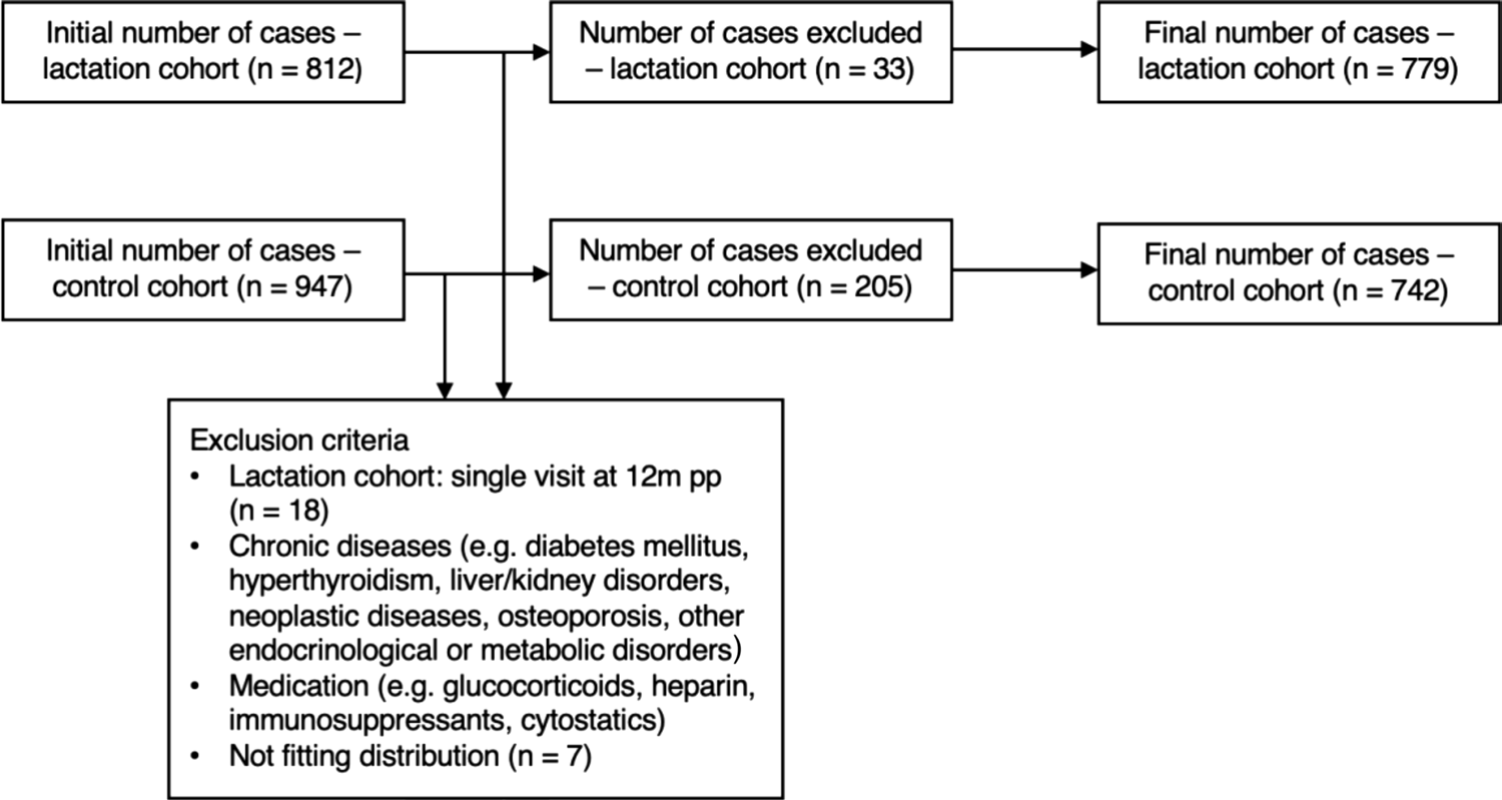
Selection of participants from the lactation and control cohorts. Flowchart showing the exclusion of individuals

The data originated from the large “LIFE Child” study, which was initiated in 2011 in Leipzig, Germany. It is part of the “Leipzig Research Centre for Civilization Diseases (LIFE)” and is aimed at monitoring children’s development, growth, and healthiness while considering various lifestyle factors. To do so, children are recruited as early as the 24th week of gestation up to the age of 20 with annual follow-ups to allow for both cross-sectional and longitudinal approaches. The children’s parents are recruited as well. Details have been described previously [24, 25]. For the current retrospective cohort study, data were collected from 04/2011 to 03/2020. A total of 812 women between the ages of 21 and 44 years old who had delivered recently were examined throughout the pp period (lactation cohort). Assessments of serum bone-metabolism-related-parameters (bmrp) (ßCTX, P1NP, OC, total Ca, P, PTH, E_2_) were performed at 3, 6, and 12 m pp. As controls, we included 947 nonpregnant, nonlactating control subjects between the ages of 20 and 45 years (control cohort) from the LIFE Adult study, which is also part of the “Leipzig Research Centre for Civilization Diseases (LIFE).” Due to the small number of bf women at the 12 m examination (*n* = 7), data from this visit were not analyzed. Women who participated only at 12 m were excluded (Fig. 1). Participants with chronic diseases (e.g., diagnoses of chronic renal and hepatic diseases, hyperthyroidism, diabetes mellitus, osteoporosis, neoplastic diseases, or other endocrinological and metabolic disorders) or medication affecting bone metabolism (e.g., glucocorticoids, heparin, warfarin, immunosuppressants, except stable doses of thyroid hormone) were also excluded as shown in Fig. 1. After visual inspection, we decided to eliminate any participants who did not fit the distribution of Ca and PTH values (*n* = 7). The cut-off values were defined as mean ± 3.5 standard deviations for the Ca and PTH values. The final sample included 779 women in the lactation cohort, with 116 participating with several pregnancies. A total of 742 women in the control cohort were included after the exclusion criteria were applied (Fig. 1).

### Anthropometric measurements

Anthropometric measurements were performed by professional health care staff using standardized procedures. For the lactation cohort, we used the prepregnancy weight from their maternity log. Body mass index (BMI) was determined by weight (kilograms) divided by height (meters) squared. Participants also completed questionnaires about their medical history, lifestyle habits, and demographic characteristics.

### Breastfeeding status

At each visit (3 m and 6 m), pp women were interviewed about their infant feeding practice. They were assigned either to the exc-bf group if they had exclusively breastfed at the respective time of examination or to the nonexc-bf group if they had not exclusively breastfed anymore or had stopped breastfeeding completely at that time. For both visits, exc-bf women and nonexc-bf women were compared with each other and with the nonpregnant, nonlactating control group. The numbers of women who participated from the lactation cohort were 618 at 3 m and 590 at 6 m (Table 1). Controls were set as the reference for the comparisons exc-bf versus controls and nonexc-bf versus controls. Nonexc-bf women were used as the reference group for the comparison exc-bf versus nonexc-bf.

**Table 1:** Anthropometric and breastfeeding characteristics of the lactation and control cohorts

Lactation cohort					Control cohort		
N (data)			Age in years (mean ± SD)	BMI in kg/m^2^ (mean ± SD)	N (data)	Age in years (mean ± SD)	BMI in kg/m^2^ (mean ± SD)
779 (N total)			31.9 (4.27)	22.7 (3.44)	742	39.2 (6.52) ***	24.4 (4.56) ***
Visit	Subgroup						
	Exc-bf	Nonexc-bf					
3 m	570	48					
6 m	360	230					

Descriptives are presented as counts for categorical variables and as means (SD) for continuous variables (age, BMI). The lactation cohort was set as the statistical reference. The means of the controls were significantly different from those of the lactation cohort: ***p < .001. *exc-bf* exclusive breastfeeding, *nonexc-bf* nonexclusive breastfeeding, *BMI* body mass index, *SD* standard deviation, *m* months

### Laboratory measurements

Venous blood samples were collected in the morning (7:30 a.m. to 10:00 a.m.) under fasting conditions. Serum parameters were measured at the Institute of Laboratory Medicine, Clinical Chemistry, and Molecular Diagnostics of the University of Leipzig, using the following methods for routine patient diagnostics: Serum samples P1NP, ßCTX, OC, and intact PTH were measured with electrochemiluminescence assays (ECLIA; Elecsys, Cobas 801, Roche Diagnostics, Mannheim, Germany). The mean interassay coefficient of variation for these four parameters was between 1.95 and 13.10% from 4 QC cycles across the representative 4 months (82–164 runs). Details can be found in Geserick et al. [26]. Total serum Ca and P were assessed with a Roche Cobas C701 analyzer (Roche Diagnostics GmbH, Mannheim, Germany). E2 was quantified by liquid chromatography-tandem mass spectrometry (LC–MS/MS). A detailed description of the method can be found in Gaudl et al. [27]. Reference levels were chosen according to the manufacturer and refer to nonpregnant, nonlactating women. We used reference levels for E2 from Bae et al. [28].

### Statistical analysis

Descriptive statistics were given as the mean and standard deviation for continuous variables and counts for categorical variables, stratified by bf status. Bmrp levels were compared between groups at each visit using simple linear regression (univariate analyses). Subsequently, we evaluated the association between bmrp levels and bf status using linear mixed effect model. These associations were corrected for age and BMI. The subject was added as random intercept to account for multiple measurements per woman. All effect sizes are given as group differences (ß). The number of cases we included varied slightly between models because of missing values in the parameters (Suppl. Table 1) [29]. Means and standard deviations for the respective parameter, cohort and visit are presented in Supplemental Table 2 [29]. Statistical analyses were conducted with R, version 4.0.5. Ggplot2 was used to create figures. The significance level was set to α = 0.05.

## Results

### Study population

Anthropometric and breastfeeding characteristics are presented in Table 1. The final sample consisted of 1,521 participants divided into a lactation cohort (*n* = 779) and controls (*n* = 742). The majority of the lactation cohort were still breastfeeding exclusively at 3 m pp (92.2%, *n* = 570). The proportion of exclusively breastfeeding participants decreased to 61.0% (*n* = 360) at 6 m. Mean age and BMI were significantly lower in the lactation cohort compared with controls (*p* < .001, Table 1). At 6 m pp, controls had a mean BMI (± SD) of 24.4 (4.56) kg/m^2^. BMI values were above 25 kg/m^2^ in 34.0% and above 30 kg/m^2^ in 10.3% of the control group. For the lactation cohort, exc-bf women had a mean BMI (± SD) of 22.4 (3.15) kg/m^2^ whereas nonexc-bf women had a mean BMI (± SD) of 22.8 kg/m^2^ (3.47) at 6 m. BMI values were above 25 kg/m^2^ in 13.7% (exc-bf) or 19.4% (nonexc-bf) and above 30 kg/m^2^ in 3.7% (exc-bf) or 5.7% (nonexc-bf) of the lactation cohort. Regarding BMI, there was no significant difference between exc-bf and nonexc-bf women at 6 m (β = 0.3, *p*= .652), while both groups had significantly lower BMI levels compared with the controls (β = − 1.7 [exc-bf vs. controls], β = − 1.5 [nonexc-bf vs. controls], both *ps* < 0.001).

### Bone-metabolism-related parameters and breastfeeding status

**Fig. 2:**
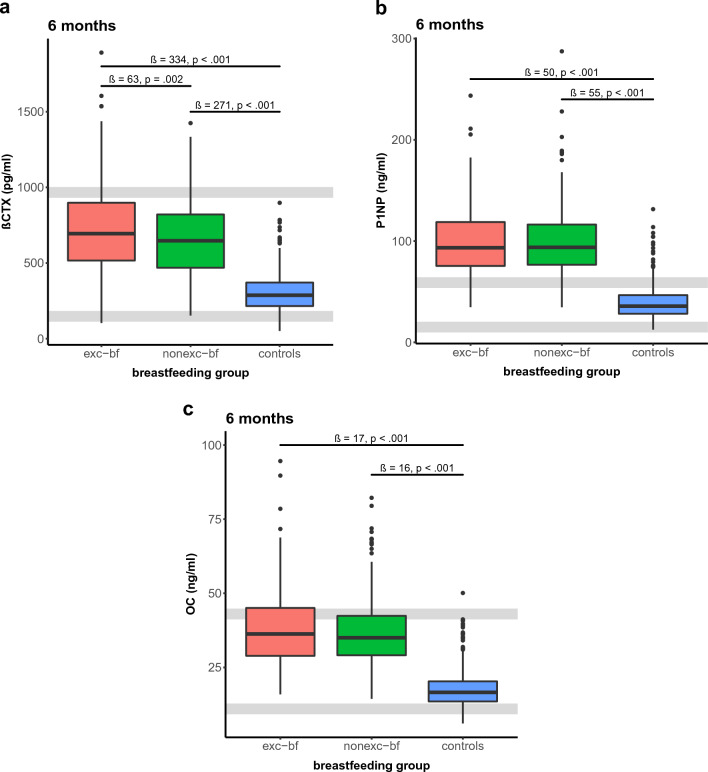
Serum ßCTX (**a**), P1NP (**b**), and OC (**c**) levels at 6 months postpartum. Exclusively breastfeeding (exc-bf) and nonexclusively breastfeeding (nonexc-bf) women were compared with controls at each visit. Results of multivariate analyses. Grey lines indicate reference levels. Bone turnover marker levels in exc-bf and nonexc-bf women were significantly higher than those of controls (all *p*s < .001) and either close to (ßCTX, OC) or above the upper reference limit (P1NP). ßCTX was significantly higher in exc-bf women than in nonexc-bf women (*p* = .002)

Results from univariate and multivariate analyses are presented in Table 2. In general, BTM levels were significantly higher in exc-bf and nonexc-bf women compared with the controls. Results were similar at 3 m and 6 m. ßCTX reached distinctly higher levels in exc-bf women (β = 419, *p* < .001 at 6 m) than in the controls in the univariate analyses. Levels in exc-bf mothers were about twice as high as those of the controls (Fig. 2a). There were also distinctly higher levels in the nonexc-bf mothers (β = 350, *p* < .001 at 6 m) than in the controls. Furthermore, the difference between nonexc-bf and exc-bf mothers was statistically significant. ßCTX levels were significantly higher in the exc-bf group by about 70 pg/ml (β = 69, *p* < .001 at 6 m) compared with nonexc-bf mothers. Effects persisted after we corrected for age and BMI (Table 2), except for the difference between nonexc-bf and exc-bf mothers at 3 m, where statistical significance was no longer achieved (β = 36, *p*= .308). For P1NP, levels in bf subjects were significantly higher by about 60 ng/ml when compared with the controls at 6 m in the univariate analyses (β = 60 [exc-bf vs. controls], β = 61 [nonexc-bf vs. controls] at 6 m, both *ps* < 0.001).

**Table 2:** Results from univariate and multivariate regression models at the 3 m and 6 m examinations

Model	Visit (m)	Comparisons	Univariate		Multivariate	
			estimate	*p*-value	estimate	*p*-value
PTH (pmol/l)	3	Exc-bf–nonexc-bf	β = − 0.4; 95% CI [− 0.8, 0]	*p* = .053	β = − 0.5; 95% CI [− 1, 0]	*p* = .049
		Exc-bf–controls	β = − 0.1; 95% CI [− 0.3, 0]	*p* = .074	β = 0; 95% CI [− 0.2, 0.3]	*p* = .647
		Nonexc-bf–controls	β = 0.3; 95% CI [− 0.1, 0.7]	*p* = .202	β = 0.6; 95% CI [0.1, 1.1]	*p* = .030
	6	Exc-bf–nonexc-bf	β = 0.3; 95% CI [0.1, 0.6]	*p* = .005	β = 0.2; 95% CI [− 0.1, 0.5]	*p* = .175
		Exc-bf–controls	β = 0.5; 95% CI [0.3, 0.7]	*p* < .001	β = 0.6; 95% CI [0.4, 0.9]	*p* < .001
		Nonexc-bf–controls	β = 0.1; 95% CI [− 0.1, 0.3]	*p* = .211	β = 0.4; 95% CI [0.1, 0.7]	*p* = .003
E_**2**_ (pmol/l)	3	Exc-bf–nonexc-bf	β = − 46; 95% CI [− 122, 30]	*p* = .233	β = − 49; 95% CI [− 197, 100]	*p* = .522
		Exc-bf–controls	β = − 124; 95% CI [− 159, − 88]	*p* < .001	β = − 150; 95% CI [− 207, − 94]	*p* < .001
		Nonexc-bf–controls	β = − 78; 95% CI [− 157, 1]	*p* = .054	β = − 102; 95% CI [− 248, 45]	*p* = .174
	6	Exc-bf–nonexc-bf	β = 12; 95% CI [− 52, 76]	*p* = .704	β = 81; 95% CI [− 76, 237]	*p* = .313
		Exc-bf–controls	β = − 82; 95% CI [− 146, − 18]	*p* = .012	β = − 55; 95% CI [− 172, 62]	*p* = .358
		Nonexc-bf–controls	β = − 95; 95% CI [− 166, − 23]	*p* = .010	β = − 135; 95% CI [− 278, 7]	*p* = .063
ßCTX (pg/ml)	3	Exc-bf–nonexc-bf	β = 77; 95% CI [13, 142]	*p* = .019	β = 36; 95% CI [33, 105]	*p* = .308
		Exc-bf–controls	β = 410; 95% CI [385, 434]	*p* < .001	β = 323; 95% CI [295, 351]	*p* < .001
		Nonexc-bf–controls	β = 332; 95% CI [268, 397]	*p* < .001	β = 287; 95% CI [219, 356]	*p* < .001
	6	Exc-bf–nonexc-bf	β = 69; 95% CI [33, 104]	*p* < .001	β = 63; 95% CI [23, 103]	*p* = .002
		Exc-bf–controls	β = 419; 95% CI [391, 446]	*p* < .001	β = 334; 95% CI [301, 367]	*p* < .001
		Nonexc-bf–controls	β = 350; 95% CI [318, 382]	*p* < .001	β = 271; 95% CI [234, 308]	*p* < .001
P1NP (ng/ml)	3	Exc-bf–nonexc-bf	β = 1; 95% CI [7, 9]	*p* = .835	β = 2; 95% CI [− 7, 10]	*p* = .711
		Exc-bf–controls	β = 53; 95% CI [50, 56]	*p* < .001	β = 43; 95% CI [39, 46]	*p* < .001
		Nonexc-bf–controls	β = 52; 95% CI [44, 60]	*p* < .001	β = 41; 95% CI [32, 50]	*p* < .001
	6	Exc-bf–nonexc-bf	β = − 1; 95% CI [− 5, 4]	*p* = .716	β = − 4; 95% CI [− 10, 1]	*p* = .094
		Exc-bf–controls	β = 60; 95% CI [56, 63]	*p* < .001	β = 50; 95% CI [46, 55]	*p* < .001
		Nonexc-bf–controls	β = 61; 95% CI [57, 65]	*p* < .001	β = 55; 95% CI [50, 60]	*p* < .001
OC (ng/ml)	3	Exc-bf–nonexc-bf	β = 1; 95% CI [− 1, 4]	*p* = .317	β = 2; 95% CI [− 1, 6]	*p* = .122
		Exc-bf–controls	β = 19; 95% CI [18, 20]	*p* < .001	β = 15; 95% CI [14, 17]	*p* < .001
		Nonexc-bf–controls	β = 17; 95% CI [14, 20]	*p* < .001	β = 13; 95% CI [10, 16]	*p* < .001
	6	Exc-bf–nonexc-bf	β = 1; 95% CI [0, 3]	*p* = .140	β = 0; 95% CI [− 2, 2]	*p* = .972
		Exc-bf–controls	β = 21; 95% CI [19, 22]	*p* < .001	β = 17; 95% CI [15, 18]	*p* < .001
		Nonexc-bf–controls	β = 19; 95% CI [18, 21]	*p* < .001	β = 16; 95% CI [15, 18]	*p* < .001
Ca (mmol/l)	3	Exc-bf – nonexc-bf	β = 0.02; 95% CI [− 0.01, 0.04]	*p* = .212	β = 0.02; 95% CI [− 0.01, 0.05]	*p* = .171
		Exc-bf – controls	β = 0.06; 95% CI [0.05, 0.07]	*p* < .001	β = 0.05; 95% CI [0.04, 0.06]	*p* < .001
		Nonexc-bf–controls	β = 0.04; 95% CI [0.02, 0.07]	*p* < .001	β = 0.03; 95% CI [0, 0.06]	*p* = .088
	6	Exc-bf–nonexc-bf	β = 0; 95% CI [− 0.02, 0.01]	*p* = .741	β = 0; 95% CI [− 0.02, 0.01]	*p* = .699
		Exc-bf–controls	β = 0.03; 95% CI [0.02, 0.04]	*p* < .001	β = 0.02; 95% CI [0.01, 0.04]	*p* < .001
		Nonexc-bf–controls	β = 0.04; 95% CI [0.02, 0.05]	*p* < .001	β = 0.03; 95% CI [0.01, 0.04]	*p* < .001
P (mmol/l)	3	Exc-bf–nonexc-bf	β = 0.04; 95% CI [− 0.01, 0.08]	*p* = .085	β = 0.04; 95% CI [− 0.02, 0.1]	*p* = .189
		Exc-bf–controls	β = 0.22; 95% CI [0.2,0.23]	*p* < .001	β = 0.2; 95% CI [0.18,0.22]	*p* < .001
		Nonexc-bf–controls	β = 0.18; 95% CI [0.14,0.22]	*p* < .001	β = 0.16; 95% CI [0.1,0.22]	*p* < .001
	6	Exc-bf–nonexc-bf	β = 0.01; 95% CI [− 0.02,0.03]	*p* = .482	β = 0; 95% CI [− 0.04,0.03]	*p* = .820
		Exc-bf–controls	β = 0.16; 95% CI [0.14,0.18]	*p* < .001	β = 0.13; 95% CI [0.11,0.16]	*p* < .001
		Nonexc-bf–controls	β = 0.15; 95% CI [0.13,0.17]	*p* < .001	β = 0.14; 95% CI [0.11,0.17]	*p* < .001

BMI and age were controlled for in the multivariate analyses. Comparisons were between exclusively breastfeeding (exc-bf) women, nonexclusively bf (nonexc-bf) women, and nonpregnant, nonlactating controls. *BMI* body mass index, *m* months

Results were comparable at the 3 m examination. We found similar patterns for OC. Effects persisted after we corrected for age and BMI (Fig. 2b, c). We did not detect a difference between exc-bf and nonexc-bf mothers in the univariate or multivariate analyses for either P1NP or OC (see Table 2).

**Fig. 3:**
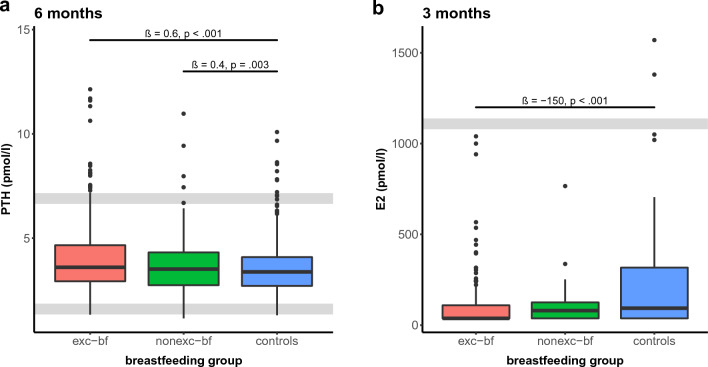
Serum PTH (**a**) and serum Estradiol (E_2_) (**b**) at the respective visit. Exclusively breastfeeding (exc-bf) and nonexclusively breastfeeding (nonexc-bf) women were compared with controls at each visit. Results of multivariate analyses. Grey lines indicate reference levels. PTH was significantly increased in exc-bf and nonexc-bf women compared with controls (*p* < .001 and *p* = .003, respectively), whereas E_2_ levels were suppressed in exc-bf and nonexc-bf women, below levels of controls (*p* < .001 and *p* = .174, respectively)

For PTH, we did not find significant differences between the groups at 3 m in the univariate analyses, whereas the levels were marginally lower in exc-bf mothers compared with nonexc-bf mothers after we adjusted for age and BMI (β = − 0.5, *p*= .049). For the multivariate analyses, nonexc-bf mothers also showed significantly higher levels than the controls (β = 0.6, *p*= .030) At 6 m, PTH reached approximately 0.3 pmol/L higher levels in exc-bf mothers (β = 0.3, *p*= .005) compared with nonexc-bf mothers and approximately 0.5 pmol/L higher levels when compared with the controls (β = 0.5, *p* < .001) in the univariate analyses. Nonexc-bf mothers showed marginally but not significantly higher levels than the controls (β = 0.1, *p*= .211). However, this effect became statistically significant after we corrected for the other predictors in the multivariate models (β = 0.4, *p*= .003). Moreover, the difference between exc-bf mothers and controls was also slightly larger after we corrected for age and BMI (β = 0.6, *p* < .001), whereas the difference between nonexc-bf and exc-bf women was no longer statistically significant (β = 0.2, *p*= .175; Fig. 3a).

For E_2_, exc-bf women had significantly lower levels compared with the controls in the univariate analyses (β = − 124, *p* < .001 at 3 m, β = − 82, *p*= .012 at 6 m). After adjusting for age and BMI, this effect remained significant only at 3 m (β = − 150, *p* < .001; Fig. 3b). The E_2_ levels of nonexc-bf women were also lower when compared with the controls; however, this effect was only significant at 6 m in the univariate analyses (β = − 95, *p*= .010). We did not find a significant difference between nonexc-bf and exc-bf mothers.

Total serum Ca and P were significantly higher in exc-bf and nonexc-bf mothers than in the controls at 3 m and 6 m in the univariate analyses. Effects persisted after we corrected for age and BMI except for the difference between nonexc-bf women and the controls at 3 m for total serum Ca levels (Table 2). There were no differences between exc-bf and nonexc-bf mothers in the univariate or multivariate models (Suppl. Figures 1 a and b) [29].

## Discussion

The aim of this study was to examine serum PTH, E_2_, BTM, Ca, and P in bf women and controls to extend the understanding of how molecular mechanisms regulate Ca metabolism during lactation. To do so, we compared bmrp levels between exc-bf and nonexc-bf women as well as healthy, nonpregnant, nonlactating controls.

In previous studies, lactational bone loss has frequently been attributed to low levels of estrogen rather than to classic calciotropic hormones including PTH and Calcitriol [6, 7, 18, 20, 30–34]. For PTH, in earlier studies, either no difference was found between bf and nonbf women or controls (a finding that is consistent with our finding at 3 m when we compared exc-bf women with controls) [15–17], or else the hormone was suppressed in lactating women [11, 18–20, 35]. Although our data showed significantly higher PTH levels in exc-bf and nonexc-bf women when compared with the controls at 6 m, we believe that this effect has only minor clinical and biological relevance, as the distributions of data in all three groups existed largely in the lower half of the reference range of the assay (Fig. 3a). Therefore, we surmise that lactational bone loss is relatively independent of PTH [36]. With respect to E_2_, lactation causes hypoestrogenemia by suppressing the hypothalamic-pituitary-ovarian axis. In agreement with other authors [6, 11, 13], our results supported this effect, as we found suppressed levels in bf women compared with controls. The difference was significant only for exc-bf women at 3 m, most likely due to the smaller case number with available E_2_ values in the exc-bf group at 6 m (Suppl. Table 1) [29]. Although the findings were not significant, E_2_ levels were also suppressed in the nonexc-bf women, a finding that could be attributed to the effect of partial bf on the hypothalamic-pituitary-ovarian axis.

During lactation, bone turnover is increased as reflected by high rates of osteoclast-driven bone resorption and osteoblast-driven bone formation [5–7, 12, 13, 16, 21, 23, 37]. Similarly, our study demonstrates elevated rates of bone resorption markers. In exc-bf women, we found increased ßCTX levels that were twice as high as those of nonpregnant, nonlactating controls, confirming prior findings by Carneiro et al. [13]. In our data, ßCTX was also higher in exc-bf women when compared with nonexc-bf women, but this difference was significant only at 6 m which might once again reside in the smaller sample size of the nonexc-bf women at 3 m (Suppl. Table 1) [29].

Similar to findings from previous papers [9, 13, 15, 20–22], our bone formation markers, assessed as OC and P1NP, were significantly elevated in exc-bf women when compared with the controls. Both OC and P1NP levels were either at the upper limit (OC) of the reference range or even considerably above it (P1NP). The substantially elevated rates of bone formation and bone resorption markers in exc-bf women support the concept of high bone turnover during lactation.

We also observed increased bone formation markers (P1NP, OC) in nonexc-bf women with levels significantly above those of the controls and almost equal to those of exc-bf women. We surmise that this finding is due to the effect of partial bf. However, the significantly lower levels of ßCTX in nonexc-bf compared with exc-bf women indicate a decrease in bone resorption that might be attributable to reduced bf as Kent et al. and others reported a normalization of bone resorption in weaning mothers [2, 15]. Nevertheless, we cannot completely rule out the possibility that the elevation in BTM in the lactation cohort was due to the effect of a previous pregnancy, as we did not have a pregnant nonbf control group, and bone turnover has been shown to also be increased in late pregnancy [9, 21, 38–40]. Further research is needed to compare bmrp in exc-bf and nonexc-bf women as well as in nonbf pregnant women.

Attributable to skeletal Ca mobilization, serum total Ca was significantly increased in exc-bf and nonexc-bf women compared with the controls but remained within normal limits. Previous studies have also reported increased or normal levels [13, 17, 19–21, 31]. Results were similar for serum P. Increased bone resorption, along with decreased renal P excretion, is thought to be the cause of increased serum P levels in lactating women, as reported by others [2].

This study has some advantages and limitations. The greatest strength of the present study was that we had large-sized lactation and control cohorts. To our knowledge, this is the largest published data that describes bone turnover in the comparison of breastfeeding and non-breastfeeding women. We were thereby able to provide an up-to-date overview on bone metabolism during lactation and re-evaluate the findings of other studies, some of which have been contradictory and have only been conducted in small samples [15, 17, 19, 21, 32, 41–43]. However, we lack measurements of Prolactin, PTHrP, Calcitonin and Calcitriol, which are also involved in bone turnover in animal or human models [2]. Missing data on other factors potentially affecting bone metabolism, such as menstrual status, breast milk volume, or Ca and P intake, must be considered when interpreting our results. In addition, we did not measure bone mineral density, so changes in BTM could not be directly correlated with bone loss. Furthermore, the number of cases we had in our lactation cohort varied by visit and lactation subgroup. In particular, the size of the group of nonexc-bf women at 3 m was comparatively small (Table 1), as the World Health Organization (WHO) recommends exclusive bf for 6 m [44]. Moreover, for the nonexc-bf group, we did not have any information about the frequency of bf. Both must be considered when interpreting our results. Lastly, the lactation and control cohorts differed significantly in BMI and age. The control cohort was significantly older and heavier. Consequently, we cannot completely rule out that the differences in bmrp levels between the lactation and control cohorts are related to the higher age and BMI of the controls. To summarize, this study shows that bone formation and bone resorption are substantially higher in exc-bf and nonexc-bf women compared with nonpregnant, nonlactating controls, indicating high bone turnover during lactation. Our data suggest that lactational bone loss is relatively independent of PTH as the data distribution of exc-bf, nonexc-bf, and control groups were in the underpart of the reference range.

### Supplementary Information

Below is the link to the electronic supplementary material.

**Suppl. Fig. 1** Serum total Ca (**a**) and serum P (**b**) at 6 months postpartum. Exclusively breastfeeding (exc-bf) and nonexclusively breastfeeding (nonexc-bf) mothers were compared with controls at each visit. Results of multivariate analyses. Grey lines indicate reference levels. Total Ca and P levels were significantly higher in exc-bf and nonexc-bf mothers compared with controls (all *p*s < .001). (PDF 15 kb)

Supplementary file 2 (PDF 16 kb)

**Suppl. Table 1** Case numbers of the lactation and control cohorts depending on the parameter measured. (PDF 122 kb)

**Suppl. Table 2** Means (SD) are presented for the respective parameter, cohort, and visit. (PDF 92 kb)

## Data Availability

Some or all datasets generated during and/or analyzed during the current study are not publicly available but are available from the corresponding author on reasonable request.
