# Supplementary material for: Bone turnover in lactating and nonlactating women
Source: Arch Gynecol Obstet. 2023 Sep 14;308(6):1853–62. doi: 10.1007/s00404-023-07189-0 (PMC10579129; doi:10.1007/s00404-023-07189-0)
Supplement: Supplementary file 1 — Suppl. Fig. 1 Serum total Ca (a) and serum P (b) at 6 months postpartum. Exclusively breastfeeding (exc-bf) and nonexclusively breastfeeding (nonexc-bf) mothers were compared with controls at each visit. Results of multivariate analyses. Grey lines indicate reference levels. Total Ca and P levels were significantly higher in exc-bf and nonexc-bf mothers compared with controls (all ps < .001). (PDF 15 kb) [file 404_2023_7189_MOESM1_ESM.pdf]

Supplemental material: Suppl. Figure 1a

Article title: Bone turnover in lactating and nonlactating women

Journal: Archives of Gynaecology and Obstetrics

Authors: Lena Nerius[1], Mandy Vogel, Uta Ceglarek, Wieland Kiess, Ronald Biemann, Holger Stepan, Jürgen Kratzsch

[1]Corresponding author; LIFE Leipzig Research Center for Civilization Diseases, University of Leipzig, 04103 Leipzig, Germany; E-Mail: lena-nerius@web.de

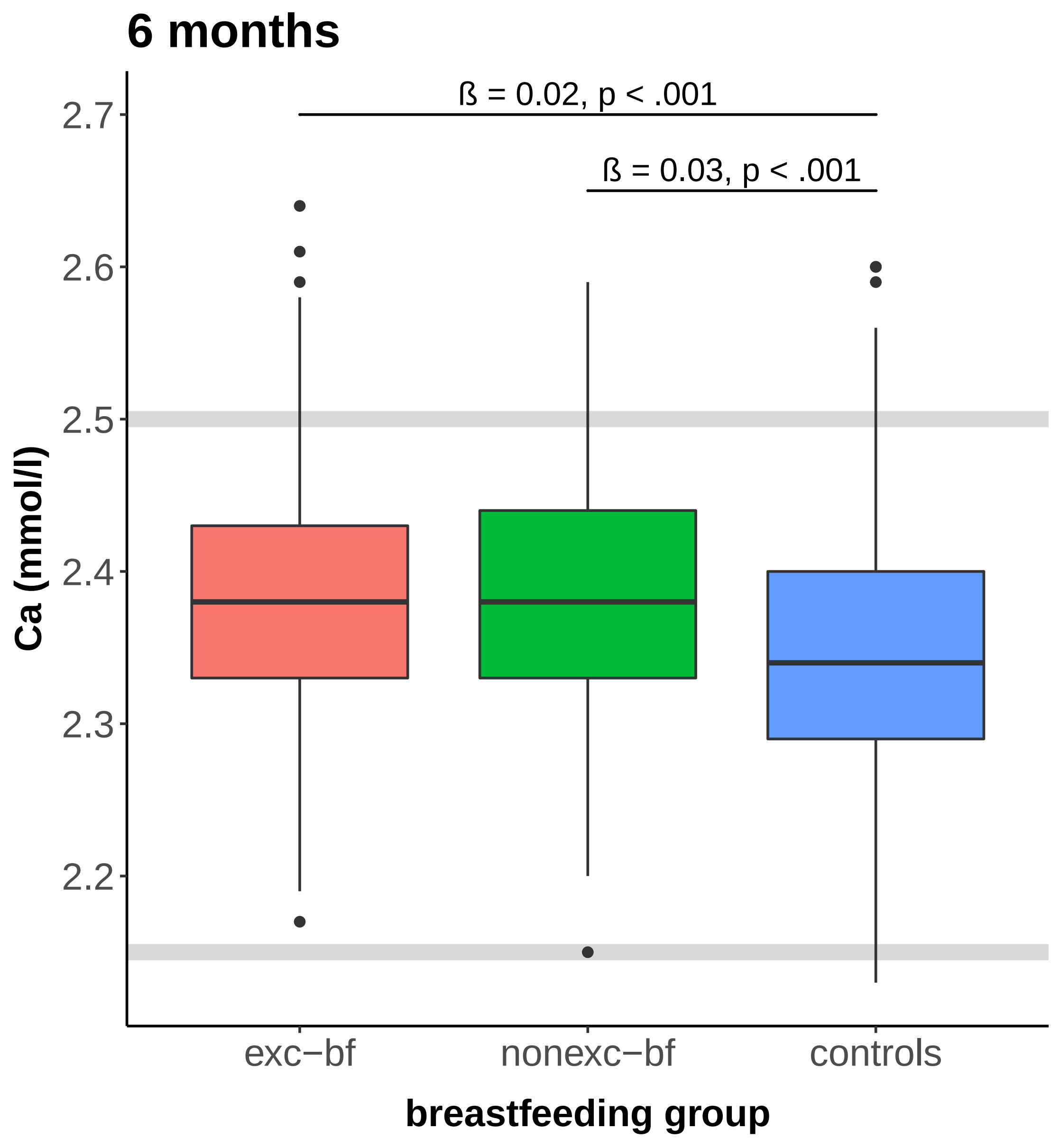
6 months
ß = 0.02, p < .001
ß = 0.03, p < .001
2.7
2.6
2.5
2.4
2.3
2.2
Ca (mmol/l)
exc−bf
nonexc−bf
controls
breastfeeding group
