# Supplementary material for: Bone turnover in lactating and nonlactating women
Source: Arch Gynecol Obstet. 2023 Sep 14;308(6):1853–62. doi: 10.1007/s00404-023-07189-0 (PMC10579129; doi:10.1007/s00404-023-07189-0)
Supplement: Supplementary file 3 — Suppl. Table 1 Case numbers of the lactation and control cohorts depending on the parameter measured. (PDF 122 kb) [file 404_2023_7189_MOESM3_ESM.pdf]

Supplemental material: Suppl. Table 1

Article title: Bone turnover in lactating and nonlactating women

Journal: Archives of Gynaecology and Obstetrics

Authors: Lena Nerius[1], Mandy Vogel, Uta Ceglarek, Wieland Kiess, Ronald Biemann, Holger Stepan, Jürgen Kratzsch

[1]Corresponding author; LIFE Leipzig Research Center for Civilization Diseases, University of Leipzig, 04103 Leipzig, Germany; E-Mail: lena-nerius@web.de

	N lactation cohort				N control cohort
	3m		6m		
	**exc-bf**	**nonexc-bf**	**exc-bf**	**nonexc-bf**	
**PTH**	542	43	348	224	626
**Estradiol**	324	21	212	127	126
**ßCTX**	540	43	364	222	624
**P1NP**	538	43	346	223	624
**OC**	541	43	347	223	624
**Ca**	571	48	360	232	725
**P**	571	48	360	232	725
