# Supplementary material for: Bone turnover in lactating and nonlactating women
Source: Arch Gynecol Obstet. 2023 Sep 14;308(6):1853–62. doi: 10.1007/s00404-023-07189-0 (PMC10579129; doi:10.1007/s00404-023-07189-0)
Supplement: Supplementary file 4 — Suppl. Table 2 Means (SD) are presented for the respective parameter, cohort, and visit. (PDF 92 kb) [file 404_2023_7189_MOESM4_ESM.pdf]

Supplemental material: Suppl. Table 2

Article title: Bone turnover in lactating and nonlactating women

Journal: Archives of Gynaecology and Obstetrics

Authors: Lena Nerius[1], Mandy Vogel, Uta Ceglarek, Wieland Kiess, Ronald Biemann, Holger Stepan, Jürgen Kratzsch

[1]Corresponding author; LIFE Leipzig Research Center for Civilization Diseases, University of Leipzig, 04103 Leipzig, Germany; E-Mail: lena-nerius@web.de

		3 m	6 m
PTH (pmol/l)	exc-bf	3.40 (1.51)	4.01 (1.69)
	nonexc-bf	3.81 (1.60)	3.68 (1.38)
	controls	3.54 (1.19)	
Estradiol (pmol/l)	exc-bf	89.0 (118)	130 (352)
	nonexc-bf	135 (168)	118 (178)
	controls	213 (263)	
ßCTX (pg/ml)	exc-bf	714 (276)	724 (283)
	nonexc-bf	637 (232)	655 (257)
	controls	305 (122)	
P1NP (ng/ml)	exc-bf	92.1 (33.2)	99.0 (32.3)
	nonexc-bf	91.3 (38.3)	99.8 (34.9)
	controls	39.2 (15.8)	
OC (ng/ml)	exc-bf	36.5 (12.1)	38.2 (12.4)
	nonexc-bf	35.0 (9.98)	37.0 (11.9)
	controls	17.6 (5.85)	
Ca (mmol/l)	exc-bf	2.41 (0.08)	2.38 (0.07)
	nonexc-bf	2.39 (0.09)	2.39 (0.08)
	controls	2.35 (0.08)	
P (mmol/l)	exc-bf	1.32 (0.14)	1.26 (0.13)
	nonexc-bf	1.28 (0.16)	1.25 (0.13)
	controls	1.10 (0.15)	
